# A Rare Case of Preeclampsia-Associated Syndrome of Inappropriate Antidiuretic Hormone Secretion (SIADH) in Twin Pregnancy Treated With Tolvaptan

**DOI:** 10.7759/cureus.115405

**Published:** 2026-08-29

**Authors:** Nathan Gilmore, Daniel Colome, Nicholas A Smith, Emery Perrin, Rolando Gomez

**Affiliations:** 1 Medicine, Charles E. Schmidt College of Medicine, Florida Atlantic University, Boca Raton, USA; 2 Neurological Surgery, Charles E. Schmidt College of Medicine, Florida Atlantic University, Boca Raton, USA; 3 Psychiatry, Charles E. Schmidt College of Medicine, Florida Atlantic University, Boca Raton, USA; 4 Medicine, Herbert Wertheim College of Medicine, Florida International University, Miami, USA; 5 Obstetrics and Gynecology, Memorial Regional Hospital, Hollywood, USA

**Keywords:** hyponatremia, nephrology, obstetrics, tolvaptan treatment, twin gestation

## Abstract

Severe hyponatremia is uncommon in pregnancy but has been described in association with preeclampsia, particularly in severe disease and multifetal gestation. We report a 38-year-old gravida 1 para 0 with dichorionic-diamniotic twins who presented at 29 weeks and four days of gestation and developed rapidly progressive hypotonic hyponatremia, with serum sodium decreasing from 126 to 110 mmol/L. Low serum osmolality with inappropriately concentrated urine, elevated urine sodium, normal thyroid testing, preserved renal function, and the initial clinical volume assessment supported a pattern consistent with syndrome of inappropriate antidiuretic hormone secretion (SIADH); however, cortisol was not obtained during the hospitalization, so adrenal insufficiency was not completely excluded biochemically. Fluid restriction, hypertonic saline, and furosemide were limited by pulmonary edema, and persistent severe hyponatremia prompted multidisciplinary use of tolvaptan despite limited pregnancy safety data. Cesarean delivery was subsequently performed in the setting of worsening maternal disease. Maternal serum sodium normalized postpartum without documented neurologic complications, supporting a pregnancy-mediated disturbance of water balance. This case highlights the diagnostic overlap between preeclampsia-associated hyponatremia and SIADH, the narrow therapeutic margin created by pulmonary edema, and the importance of close sodium monitoring and timely delivery when maternal status deteriorates.

## Introduction

Normal pregnancy resets osmotic regulation early in gestation. The thresholds for thirst and arginine vasopressin release decrease, producing an approximately 3-5 mmol/L fall in maternal serum sodium and a 5-10 mOsm/kg reduction in plasma osmolality compared with the nonpregnant state [1-3]. These changes support physiologic plasma-volume expansion, but they create an important diagnostic pitfall as follows: mild hyponatremia may be normal in pregnancy, whereas serum sodium below approximately 130 mmol/L, particularly values approaching 110 mmol/L, is not explained by the normal gestational reset alone [2,3]. Severe hypotonic hyponatremia is clinically important because maternal cerebral edema, seizures, and other neurologic complications may occur, while maternal-fetal sodium equilibration can expose the fetus and neonate to the same osmotic disturbance [3].

The association between preeclampsia and pathologic hyponatremia is increasingly recognized. In 332 pregnancies complicated by preeclampsia, Razavi et al. identified hyponatremia in 9.7%, with higher frequency among patients with severe features and twin gestations [4]. A systematic review of 55 reported cases found multifetal gestation in 34% and described both syndrome of inappropriate antidiuretic hormone secretion (SIADH) and hypervolemic mechanisms [5]. Subsequent cohorts have reinforced the relationship between lower sodium and disease severity as follows: Remer et al. reported hyponatremia in 14.6% of 700 patients with preeclampsia [6], Endres et al. found greater use of emergency antihypertensives, antenatal corticosteroids, and cesarean delivery among hyponatremic patients with severe features [7], and Morton et al. reported frequent fetal growth restriction, acute kidney injury, postpartum intensive care admission, and urgent delivery in moderate to severe cases [8]. These data suggest that hyponatremia may accompany the broader maternal and placental dysfunction of severe preeclampsia rather than represent an isolated electrolyte abnormality.

Published case reports also demonstrate substantial mechanistic heterogeneity. Jhaveri et al. described preeclampsia with hyponatremia and oliguria in a twin pregnancy that remitted after cesarean delivery [9], Hinkson et al. reported severe preeclampsia-associated hyponatremia attributed to inappropriate antidiuretic hormone secretion [10], and Pu et al. identified 21 prior cases in addition to their own, with most reported patients improving soon after delivery [11]. Against this background, we report a dichorionic-diamniotic twin pregnancy complicated by profound hypotonic hyponatremia with an SIADH-like biochemical pattern, pulmonary edema that constrained conventional therapy, rescue use of tolvaptan, and postpartum normalization after cesarean delivery.

## Case presentation

A 38-year-old gravida 1 para 0 with a dichorionic-diamniotic twin pregnancy, gestational hypertension, advanced maternal age, migraines, and vertigo presented at 29 weeks and four days of gestation for antenatal corticosteroids. The pregnancy was complicated by fetal growth restriction and absent end-diastolic flow in twin A. On arrival, she was contracting without leakage of fluid or vaginal bleeding and was transferred to a higher-acuity center because of concern for possible preterm delivery. Initial blood pressure was 143/78 mmHg, oxygen saturation was 98%, and examination demonstrated a gravid, nontender abdomen, trace bilateral lower-extremity edema, unlabored respirations, and cervical dilation of 1 cm.

Initial laboratory evaluation demonstrated true hypotonic hyponatremia and is summarized with pregnancy-specific reference intervals in Table 1. Serum sodium was 126 mmol/L with serum osmolality of 253 mOsm/kg and subsequently declined to a nadir of 110 mmol/L; glucose of 93 mg/dL was not sufficient to account for the hyponatremia through a hyperglycemia-mediated translocational mechanism. Urine osmolality was 420 mOsm/kg and urine sodium was 62 mmol/L. Thyroid-stimulating hormone was 1.6 mIU/L and within the pregnancy-specific reference range; cortisol was not obtained during the hospitalization. In the setting of preserved renal function and the initial clinical volume assessment, the combination of hypotonicity with inappropriately concentrated urine and elevated urine sodium was interpreted as an SIADH-like biochemical pattern (Table 1) [3,12]. Because cortisol was not measured, adrenal insufficiency could not be completely excluded biochemically.

**Table 1:** Initial laboratory evaluation and relevant reference intervals. Reference intervals are representative third-trimester pregnancy ranges where available and may vary by assay and laboratory. Random urine osmolality and urine sodium are context-dependent and therefore are presented using SIADH interpretive thresholds rather than fixed pregnancy reference interval. AST: aspartate aminotransferase; ALT: alanine aminotransferase; SIADH: syndrome of inappropriate antidiuretic hormone secretion

Parameters	Patient result	Reference range or interpretive threshold	Interpretation
Serum sodium, initial	126 mmol/L	130-148 mmol/L (third trimester) [2]	Low
Serum sodium, nadir	110 mmol/L	130-148 mmol/L (third trimester) [2]	Profoundly low
Serum sodium, subsequent 48 h	117-127 mmol/L	130-148 mmol/L (third trimester) [2]	Persistent hyponatremia
Serum osmolality	253 mOsm/kg	278-280 mOsm/kg (third trimester) [2]; <275 mOsm/kg indicates hypotonicity [12]	Low; confirms hypotonicity
Glucose	93 mg/dL	71-77 mg/dL fasting (third trimester) [2]; fasting status was not documented	Not sufficiently elevated to explain hyponatremia
Creatinine	0.83 mg/dL	0.4-0.9 mg/dL (third trimester) [2]	Within pregnancy-specific interval
AST	37 U/L	4-32 U/L (third trimester) [2]	Mildly above pregnancy-specific interval
ALT	33 U/L	2-25 U/L (third trimester) [2]	Mildly above pregnancy-specific interval
Albumin	3.4 g/dL	2.3-4.2 g/dL (third trimester) [2]	Within pregnancy-specific interval
Urine osmolality	420 mOsm/kg	≤100 mOsm/kg expected with appropriate vasopressin suppression during hypotonic hyponatremia; >100 mOsm/kg is inappropriately concentrated [12]	Inappropriately concentrated; supports persistent antidiuretic activity
Urine sodium	62 mmol/L	No fixed random reference interval; >30 mmol/L supports SIADH in euvolemic hypotonic hyponatremia with normal salt intake [12]	Elevated; supports an SIADH pattern
Thyroid-stimulating hormone	1.6 mIU/L	0.3-3.0 mIU/L (third trimester) [2]	Within normal third-trimester range; thyroid dysfunction not supported
Cortisol	-	12-50 µg/dL (third trimester) [2]	Value not obtained during hospital visit

Management began with fluid restriction and close serum sodium monitoring. Given the severity of the hyponatremia, hypertonic saline and furosemide were used. The patient subsequently developed pulmonary edema with dyspnea and hypoxemia requiring supplemental oxygen, which reduced tolerance for additional sodium-containing fluid. Because severe hyponatremia persisted despite conventional therapy, tolvaptan was initiated after multidisciplinary discussion of the potential maternal benefit and the limited pregnancy safety data. Serum sodium improved after tolvaptan initiation, and treatment was continued for four days.

Cesarean delivery was performed in the setting of worsening maternal hyponatremia and preeclampsia in a twin gestation. Two liveborn neonates were delivered without documented morphologic abnormalities in the available case record. Maternal serum sodium normalized postpartum, and no maternal neurologic complications were documented. The postpartum resolution supported a pregnancy-mediated disturbance in antidiuretic water handling.

## Discussion

The central diagnostic problem in this case was not distinguishing normal pregnancy from abnormal sodium physiology; a nadir serum sodium of 110 mmol/L is far beyond the modest gestational reduction produced by the reset osmostat [1-3]. The more difficult distinction was between SIADH and dilutional, hypervolemic hyponatremia related to preeclampsia. Our patient's hypo-osmolality, inappropriately concentrated urine, elevated urine sodium, preserved renal function, and normal thyroid testing supported an SIADH-like biochemical pattern [12]. Cortisol was not obtained, however, so adrenal insufficiency could not be completely excluded biochemically, and a definitive SIADH diagnosis cannot be established from the available data. Yet preeclampsia can produce total-body fluid excess while effective arterial circulating volume remains reduced, allowing nonosmotic vasopressin release despite edema. This explains why subsequent pulmonary edema does not necessarily refute the initial SIADH-like biochemical phenotype. Powel et al. found the same diagnostic overlap across 55 published preeclampsia-associated cases as follows: when a mechanism was assigned, 41% were classified as SIADH and 59% as hypervolemic hyponatremia [5]. The present case therefore likely lies along a physiologic spectrum rather than fitting a purely euvolemic or hypervolemic category throughout the hospitalization.

The closest published cases also strengthen the possibility of a pregnancy- and placental-mediated driver. Powel et al. described a 45-year-old patient with a dichorionic twin gestation, preeclampsia, and sodium of 125 mmol/L whose hyponatremia persisted despite water restriction and furosemide but resolved within 48 h after delivery [5]. Jhaveri et al. likewise reported a twin pregnancy complicated by preeclampsia, hyponatremia, and oliguria with complete remission after cesarean delivery [9], while Hinkson et al. specifically attributed severe preeclampsia-associated hyponatremia to inappropriate antidiuretic hormone secretion [10]. Our patient shared twin gestation and postpartum resolution but presented earlier and with substantially greater biochemical severity, reaching 110 mmol/L and developing pulmonary edema that limited conventional therapy. Pu et al.'s review of 22 cases found that 15 patients recovered from hyponatremia within 72 h of delivery [11]. More recently, Horsburgh and Lazzaro described early-onset preeclampsia at 24+4 weeks with hypervolemic hyponatremia to 118 mmol/L that worsened despite fluid restriction and normalized by postpartum day four after emergency cesarean delivery and perioperative hypertonic saline [13]. Together, these cases suggest that delivery can act as a physiologic dechallenge as follows: rapid postpartum improvement supports removal of a pregnancy-related stimulus, although it cannot identify whether placental vasopressin handling, reduced effective circulating volume, or another mechanism predominated.

The sodium abnormality may also be a marker of the severity of the underlying preeclamptic process rather than an isolated electrolyte complication. Razavi et al. observed hyponatremia in 9.7% of 332 preeclamptic pregnancies and found it more frequently in severe disease and twin gestations [4]. Remer et al. reported a prevalence of 14.6% among 700 patients with preeclampsia and an association with severe features and adverse obstetric and neonatal outcomes [6]. In a cohort limited to preeclampsia with severe features, Endres et al. found that patients with sodium below 134 mmol/L more often required emergency antihypertensive therapy, antenatal corticosteroids, and cesarean delivery [7]. Morton et al. similarly reported fetal growth restriction in 36.4%, acute kidney injury in 34.1%, postpartum intensive care admission in 28.7%, and urgent delivery within 24 h in 71% of women with moderate-to-severe hyponatremia [8]. Our patient's twin gestation, fetal growth restriction with absent end-diastolic flow, pulmonary edema, antenatal corticosteroid administration, and eventual cesarean delivery align with this higher-risk phenotype. These associations do not prove that hyponatremia drives preeclampsia severity, but they support treating a falling sodium concentration as a potential marker of worsening maternal-placental physiology that warrants intensified surveillance.

Treatment in this case was constrained by competing risks. Fluid restriction is the conventional initial strategy for SIADH, while hypertonic saline is used when hyponatremia is profound or neurologically threatening [3,12]. Once pulmonary edema developed, however, further saline-based correction risked worsening respiratory compromise. Tolvaptan offered a mechanistically different approach because selective V2-receptor antagonism produces aquaresis and raises serum sodium without requiring an additional sodium-containing fluid load [12,14]. In nonpregnant adults, the prescribing information supports tolvaptan for clinically significant euvolemic or hypervolemic hyponatremia, including SIADH, but requires inpatient initiation and close sodium monitoring because overly rapid correction can cause osmotic demyelination [14]. Pregnancy introduces a separate concern as follows: available human data are insufficient to define developmental risk, and animal studies have demonstrated fetal toxicity at maternally toxic exposures [14]. A 2025 report by Dong et al. provides a useful but importantly different comparator as follows: a pregnant patient with cervical large-cell neuroendocrine carcinoma developed severe SIADH after cesarean delivery during chemotherapy, and postpartum tolvaptan corrected refractory hyponatremia [15]. In contrast, our patient's exposure occurred before delivery in the setting of preeclampsia and pulmonary edema. Thus, the response in our case is clinically notable but should be interpreted as exceptional rescue therapy under multidisciplinary supervision, not evidence supporting routine antepartum tolvaptan use. Moreover, because tolvaptan treatment and delivery occurred within the same clinical course, the relative contribution of the drug versus removal of the pregnancy-related antidiuretic stimulus cannot be isolated.

This report has several limitations. It describes a single patient and therefore cannot establish causality, efficacy, or fetal safety. Urine osmolality and urine sodium values were available, but the timing of urine studies relative to diuretic therapy was not sufficiently granular to completely separate SIADH from overlapping preeclampsia-associated hypervolemic physiology. Direct vasopressin or copeptin measurements were not available, and detailed neonatal electrolyte values and long-term neurodevelopmental follow-up were not documented. These limitations are particularly important when interpreting the apparent response to tolvaptan. The principal contribution of this case is therefore not to establish a new standard therapy, but to illustrate how profound hyponatremia, preeclampsia, multifetal gestation, and pulmonary edema can converge to narrow conventional treatment options and require coordinated obstetric, nephrologic, and critical-care decision-making.

## Conclusions

Severe hypotonic hyponatremia should not be attributed to normal pregnancy physiology, particularly when serum sodium continues to fall in a patient with preeclampsia or multifetal gestation. An SIADH pattern is suggested by hypo-osmolality with inappropriately concentrated urine and elevated urine sodium after carefully assessing competing endocrine, renal, and volume-related causes. Management must balance neurologic risk from profound hyponatremia against the hazards of volume loading and overly rapid correction, especially when pulmonary edema is present. Delivery may remove a pregnancy-mediated antidiuretic stimulus when maternal disease worsens. Tolvaptan should be viewed as an exceptional rescue strategy rather than routine therapy in pregnancy because human fetal safety data remain insufficient.
